# Transcatheter Aortic Valve Replacement Outcomes in End-Stage Renal Disease Patients on Hemodialysis Requiring Midodrine

**DOI:** 10.1016/j.shj.2023.100163

**Published:** 2023-03-03

**Authors:** Ethan C. Korngold, Ruyun Jin, Kateri J. Spinelli, Vishesh Kumar, Brydan Curtis, Sameer Gafoor, Derek Phan, Daniel Spoon, Aidan Raney, Lisa McCabe, Brandon Jones

**Affiliations:** a Center for Cardiovascular Analytics, Research and Data Science (CARDS), Providence Heart Institute, Providence St. Joseph Health, Portland, Oregon, USA; b Providence Spokane Heart Institute, Spokane, Washington, USA; c Swedish Heart & Vascular Institute, Cherry Hill Campus, Seattle, Washington, USA; d CardioVascular Center Frankfurt, Frankfurt, Germany; e Providence International Heart Institute of Montana, Missoula, Montana, USA; f Providence St. Joseph Hospital-Orange, Orange, California, USA; g Providence Regional Medical Center Everett, Everett, Washington, USA

**Keywords:** End stage renal disease, Midodrine, Survival, Transcatheter aortic valve replacement

## Abstract

**Background:**

Patients with dialysis-dependent end-stage renal disease (ESRD) taking midodrine may be at high risk for poor outcomes following transcatheter aortic valve replacement (TAVR). We evaluated dialysis-dependent ESRD patients taking midodrine.

**Methods:**

We conducted a retrospective analysis of non-clinical trial TAVR patients from February 2012 to December 2020 from 11 facilities in a Western US health system. Patient groups included ESRD patients on midodrine before TAVR (ESRD [+M]), ESRD patients without midodrine (ESRD [−M]), and non-ESRD patients. The endpoints of 30-day and 1-year mortality were represented by Kaplan–Meier survival estimator and compared by log-rank test.

**Results:**

Forty-five ESRD (+M), 216 ESRD (−M), and 6898 non-ESRD patients were included. ESRD patients had more comorbid conditions, despite no significant difference in predicted Society of Thoracic Surgeons mortality risk between ESRD (+M) and ESRD (−M) (8.7% vs. 9.2%, *p* = 0.491). Thirty-day mortality was significantly higher for ESRD (+M) patients vs. ESRD (−M) patients (20.1% vs. 5.6%, *p* = 0.001) and for ESRD (+M) vs. non-ESRD patients (2.5%, *p* < 0.001). One-year mortality trended higher for ESRD (+M) vs. ESRD (−M) patients (41.9% vs. 29.8%, *p* = 0.07), and was significantly higher for ESRD (+M) vs. non-ESRD patients (10.7%, *p* < 0.001). Compared to ESRD (−M), ESRD (+M) patients had a higher incidence of 30-day stroke (6.7% vs. 1.4%, *p* = 0.033), 30-day vascular complications (6.7% vs. 0.9%, *p* = 0.011), and a lower rate of discharge to home (62.2% vs. 84.7%, *p* < 0.001). In contrast, ESRD (−M) patients had no significant differences from non-ESRD patients for these outcomes.

**Conclusions:**

Our experience suggests ESRD patients on midodrine are a higher acuity population with worse survival after TAVR, compared to ESRD patients not on midodrine. These findings may help with risk stratification for ESRD patients undergoing TAVR.

## Introduction

Registry data have demonstrated that transcatheter aortic valve replacement (TAVR) patients with end-stage renal disease (ESRD) on hemodialysis have higher in-hospital and 1-year mortality than nondialysis-dependent patients and non-ESRD patients.^1^, ^2^, ^3^ Anecdotal evidence from our programs has suggested that the subgroup of patients with ESRD on dialysis taking the oral medication midodrine may be at particularly high risk for poor outcomes following TAVR. Midodrine is an alpha-1 adrenergic receptor agonist which induces arterial and venous vasoconstriction.^4^ It is FDA-approved for the treatment of symptomatic orthostatic hypotension and has been shown to improve intradialytic hypotension in dialysis-dependent ESRD patients.^5^

ESRD dialysis-dependent patients taking midodrine with aortic stenosis are often referred for TAVR specifically to address intradialytic hypotension and the inability to tolerate dialysis. Predicting which patients may benefit from TAVR is of great clinical significance. We aimed to compare outcomes for ESRD dialysis-dependent TAVR patients stratified by those taking midodrine and those not taking midodrine, as well as compared to non-ESRD patients.

## Methods

### Patient Population and Study Endpoints

We retrospectively analyzed data that were collected according to the Society of Thoracic Surgery/American College of Cardiology Transcatheter Valve Therapy (TVT) registry specifications.^6^ All nonclinical trial TAVR procedures performed between February 1, 2012 and December 31, 2020 at 11 facilities in the Providence St. Joseph Health system were included. ESRD dialysis-dependence was defined as patients who are currently undergoing either hemodialysis or peritoneal dialysis on an ongoing basis as a result of renal failure, according to the registry definition. Midodrine status at the time of the procedure was captured through chart review. This study was approved by the Providence St. Joseph Health institutional review board, with waiver of informed consent.

The primary endpoints were 30-day and 1-year mortality. Secondary endpoints were procedural outcomes, in-hospital outcomes, and 30-day cardiovascular outcomes. Patients were grouped into 3 groups as follows: ESRD dialysis-dependent patients taking midodrine prior to procedure (ESRD (+M)), ESRD dialysis-dependent patients not taking midodrine (ESRD (−M)), and patients who were not dialysis-dependent (non-ESRD).

### Statistical Analysis

Demographics and procedural characteristics were compared between groups using t-test or Wilcoxon rank-sum test for continuous variables and chi-squared or Fisher’s exact test for categorical variables. The primary endpoints of 30-day and 1-year mortality were represented by Kaplan–Meier survival estimator and compared between groups by log-rank test. Other outcomes with competing risk to mortality (30-day myocardial infarction, 30-day stroke, 30-day life-threatening or major bleeding, and 30-day major vascular complications) were represented by the cumulative incidence function, with point-wise confidence intervals (CI) obtained using the method proposed by Choudhury and compared by Gray's test.^7^^,^^8^

Missing data rates were <1% for all variables except Society of Thoracic Surgeons (STS) mortality risk for surgical aortic valve replacement (n = 80), aortic valve mean gradient (n = 167), and follow-up data. The TVT registry requires 30-day follow-up between days 21 to 75 and 1-year follow-up on day 365 ± 60 post procedure. To capture the additional survival status, electronic medical record system searching was performed. Approximately 1% of patients were missing 30-day survival status and 12% were missing 1-year survival status. Approximately 5% of patients were missing other 30-day outcomes.

The R statistical program (www.r-project.org, R Foundation for Statistical Computing, Vienna, Austria), version 4.1.2 was used for all analyses.

## Results

A total of 7159 patients were included in the study, with median (IQR) age of 81.7 (75.5-86.7) and 44.5% female. We identified 45 ESRD (+M), 216 ESRD (−M) and 6898 non-ESRD patients during the study period (Table 1). Median age was comparable between the ESRD (+M) and ESRD (−M) groups (73.3 vs. 74.6, respectively, *p* = 0.266) and higher for the non-ESRD group (81.9, *p* < 0.001). There was no significant difference in predicted STS risk of mortality between the ESRD (+M) and ESRD (−M) groups (8.7% vs. 9.2%, respectively, *p* = 0.491). Compared to ESRD (−M) patients, ESRD (+M) patients had significantly higher rates of prior coronary artery bypass graft surgery, home oxygen usage, New York Heart Association class III/IV, cardiogenic shock within 24 ​hours, and lower mean aortic valve gradients. ESRD (+M) patients had a higher rate of prior stroke than ESRD (−M) patients (22.2% vs. 13.9%), although this was not statistically significant.

**Table 1:** Patient demographics

				*p*-value		
Variable	ESRD (+M) (n = 45)	ESRD (−M) (n = 216)	Non-ESRD (n = 6898)	ESRD (+M) vs. ESRD (−M)	ESRD (+M) vs. Non-ESRD	ESRD (−M) vs. Non-ESRD
Age, years	73.3 (68.2-77.1)	74.6 (68.5-81.2)	81.9 (75.8-96.8)	0.266	<0.001	<0.001
Female	16 (35.6)	90 (41.7)	3080 (44.7)	0.448	0.221	0.384
Prior CABG	13 (28.9)	34 (15.7)	1158 (16.8)	0.037	0.031	0.682
Prior stroke	10 (22.2)	30 (13.9)	811 (11.8)	0.158	0.030	0.341
Diabetes	31 (68.9)	125 (57.9)	2429 (35.3)	0.170	<0.001	<0.001
Home oxygen	11 (24.4)	25 (11.6)	554 (8.0)	0.023	<0.001	0.061
NYHA class III or IV within 2 wk	42 (95.5)	163 (75.8)	4532 (66.0)	0.002	<0.001	0.003
Cardiogenic shock within 24 ​h	6 (13.3)	2 (0.9)	61 (0.9)	<0.001	<0.001	0.717
STS mortality risk for surgical AVR, %	8.7 (5.9-17.9)	9.2 (6.2-13.6)	4.2 (2.7-6.6)	0.491	<0.001	<0.001
AV mean gradient	38.0 (29.0-43.0)	41.0 (33.0-49.8)	40.0 (31.0-48.0)	0.014	0.077	0.079

*Notes*. Data presented as median (IQR) or n (%).

AV, aortic valve; AVR, aortic valve replacement; CABG, coronary artery bypass graft; ESRD, end-stage renal disease; M, midodrine; NYHA, New York Heart Association; STS, Society of Thoracic Surgeons.

**Figure 1:**
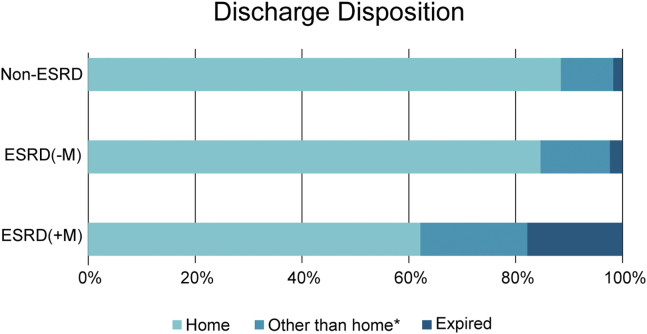
**Discharge disposition for transcatheter aortic valve replacement patients with end-stage renal disease (ESRD) on hemodialysis and midodrine (+M), ESRD on hemodialysis not on midodrine (−M), and non-ESRD patients.** ∗Other than home category includes the following: extended care/transitional care unit/rehab, other acute care hospital, nursing home, hospice, left against medical advice, or other.

Valve-in-valve procedures, anesthesia usage, and access site were similar between the 2 ESRD groups (Table 2). ESRD (+M) patients had significantly higher rates of nonelective procedures. Post-operative length of stay was similar between the 2 ESRD groups (Table 3). ESRD (+M) patients had lower rates of discharge to home, 62% compared to 85% for ESRD (−M) patients and 89% for non-ESRD patients (Figure 1).

**Table 2:** Procedural characteristics

				*p*-value		
Variable	ESRD (+M) (n = 45)	ESRD (−M) (n = 216)	Non-ESRD (n = 6898)	ESRD (+M) vs. ESRD (−M)	ESRD (+M) vs. Non-ESRD	ESRD (−M) vs. Non-ESRD
Nonelective procedure	14 (31.1)	30 (13.9)	557 (8.1)	0.005	<0.001	0.002
Valve-in-valve procedure	5 (11.1)	16 (7.4)	465 (6.7)	0.406	0.246	0.704
General anesthesia	21 (46.7)	108 (50.0)	2765 (40.1)	0.684	0.374	0.004
Valve sheath access site, femoral	42 (93.3)	190 (88.8)	6261 (91.0)	0.590	0.795	0.256

*Notes*. Data presented as n (%).

ESRD, end-stage renal disease; M, midodrine.

**Table 3:** Outcomes

				*p*-value		
Variable	ESRD (+M) (n = 45)	ESRD (−M) (n = 216)	Non-ESRD (n = 6898)	ESRD (+M) vs. ESRD (−M)	ESRD (+M) vs. Non-ESRD	ESRD (−M) vs. Non-ESRD
Postoperative length of stay, days	3.0 (2.0-6.0)	2.0 (1.0-4.0)	2.0 (1.0-3.0)	0.150	<0.001	<0.001
Discharge disposition (N (%))				<0.001	<0.001	0.238
Expired	8 (17.8%)	5 (2.3%)	117 (1.7%)			
Home	28 (62.2%)	183 (84.7%)	6105 (88.5%)			
Other than home∗	9 (20.0%)	28 (13.0%)	676 (9.8%)			
30-d mortality†	20.1 (11.0, 35.1)	5.6 (3.2, 9.6)	2.5 (2.2, 2.9)	0.001	<0.001	0.006
30-d myocardial infarction‡	0	0.5 (0.0, 2.4)	0.5 (0.3, 0.7)	0.648	0.649	0.997
30-d stroke‡	6.7 (1.7, 16.5)	1.4 (0.4, 3.7)	2.3 (1.9, 2.6)	0.033	0.049	0.394
30-d life threatening or major bleeding‡	11.2 (4.0, 22.5)	7.5 (4.5, 11.5)	6.2 (5.6, 6.8)	0.418	0.164	0.424
30-d major vascular complications‡	6.7 (1.7, 16.5)	0.9 (0.2, 3.1)	1.8 (1.5, 2.1)	0.011	0.013	0.068
1-y mortality†	41.9 (28.7, 58.1)	29.8 (24.1, 36.5)	10.7 (10.0, 11.5)	0.070	<0.001	<0.001

*Notes*. Data presented as % (95% CI), except post-operative length of stay which is presented as median (IQR).

ESRD, end-stage renal disease; M, midodrine.

∗ Discharge disposition other than home includes: extended care/transitional care unit/rehab, other acute care hospital, nursing home, hospice, left against medical advice, or other.

† Kaplan-Meier estimate.

‡ Cumulative incidence function.

**Figure 2:**
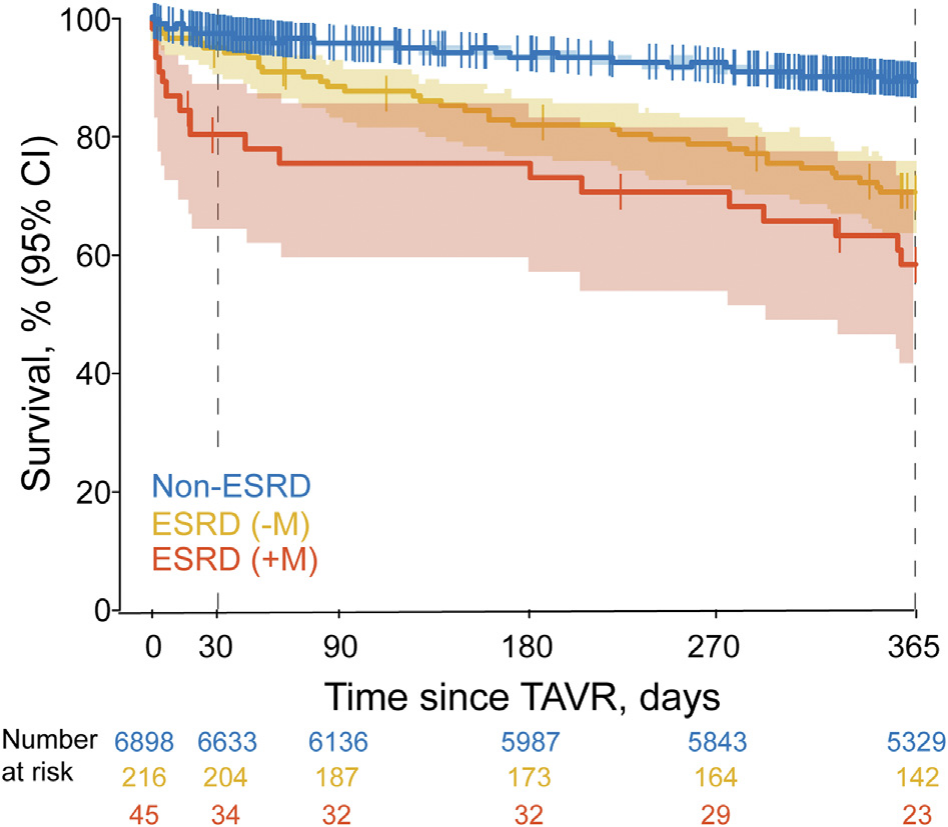
**Kaplan–Meier survival analysis of transcatheter aortic valve replacement patients with ESRD on hemodialysis and midodrine (+M) (red), ESRD on hemodialysis not on midodrine (−M) (yellow), and non-ESRD patients (blue).** Log-rank test *p*-value for the 3-group comparison <0.001. Abbreviations: CI, confidence interval; ESRD, end stage renal disease; M, midodrine; TAVR, transcatheter aortic valve replacement.

Thirty-day mortality was significantly higher for ESRD (+M) (20.1% [95% CI = 11.0%-35.1%]) compared to ESRD (−M) (5.6% [3.2%-9.6%], *p* = 0.001) and non-ESRD patients (2.5% [2.2%-2.9%], *p* < 0.001) (Table 3, Figure 2). At 1 ​year, mortality trended higher for ESRD (+M) (41.9% [28.7%-58.1%]) compared to ESRD(−M) (29.8% [24.1%-36.5%]) but was not statistically significant (*p* = 0.07) and was significantly higher than non-ESRD patients (10.7% [10.0%-11.5%]) (*p* < 0.001).

Other outcomes were similar between groups, with the exception of 30-day stroke and vascular complications (Table 3). Rates of 30-day stroke were higher in the ESRD (+M) patients (6.7% [1.7-16.5%]) compared to ESRD (−M) patients (1.4% [0.4%-3.7%], *p* = 0.033) and non-ESRD patients (2.3% [1.9%-2.6%], *p* = 0.049). Rates of 30-day vascular complications were higher in ESRD (+M) patients (6.7% [1.7%-16.5%]) compared to ESRD (−M) patients (0.9% [0.2%-3.1%], *p* = 0.011) and non-ESRD patients (1.8% [1.5%-2.1%], *p* = 0.013).

## Discussion

In our multicenter health system, ESRD dialysis-dependent patients taking midodrine at the time of TAVR had exceptionally poor outcomes. In these patients, we observed a strikingly high 20.1% mortality rate at 30-days and 41.9% mortality at 1 year. Furthermore, these patients had significantly higher health care utilization, with longer lengths of stay and only 62% discharged to home. This is especially relevant in the current health care delivery climate with staffing and resource constraints which limit patient access to care. Of particular note is that the poor outcomes in this subgroup occurred despite a similar baseline-predicted STS mortality risk compared to ESRD patients not on midodrine.

To our knowledge, prior reports of TAVR have not separated out ESRD dialysis-dependent patients taking midodrine as a particularly high-risk subgroup of ESRD dialysis-dependent patients. This variable is not collected as part of the national TVT database. Midodrine use has been associated with delayed graft function in recipients of renal transplant.^9^ It is possible that poor post-procedural outcomes may be related to peripheral vasoconstriction caused by the direct pharmacological action of midodrine. More likely, ESRD dialysis-dependent patients taking midodrine are a higher risk subgroup due to their inability to tolerate dialysis, orthostatic hypotension, or some combination thereof. This is underscored by the higher rates of comorbid conditions we observed in these patients. Furthermore, 96% of the ESRD dialysis-dependent patients taking midodrine in our study had New York Heart Association Class III/IV heart failure, which could indicate difficulty controlling volume on dialysis. We also observed that ESRD dialysis-dependent patients taking midodrine had lower mean aortic valve gradients. Previous studies have shown that low flow/low gradient aortic stenosis patients have poorer outcomes when undergoing aortic valve replacement.^10^, ^11^, ^12^, ^13^, ^14^ While our study was designed to be exploratory and hypothesis-generating, further research is needed to determine the mechanism of action.

Limitations to this study include the retrospective design and the small sample size for the ESRD subgroups. Because of the small sample size and the low event rates, we could not examine risk-adjusted or propensity-matched outcomes for the ESRD groups. We did not stratify non-ESRD patients for midodrine status, thus some patients in the non-ESRD group may have been on midodrine. Future studies examining midodrine status in all TAVR patients would be valuable for determining the role midodrine may play in adverse TAVR outcomes.

## Conclusion

Our findings suggest that TAVR outcomes are extremely poor for ESRD patients on midodrine, and extreme caution should be undertaken if offering TAVR to these patients. Further studies with larger sample sizes stratifying midodrine usage within ESRD dialysis-dependent patients undergoing TAVR may determine the degree that worse outcomes in ESRD patients are driven by midodrine usage.

## Ethics Statement

This study was approved by the Providence St. Joseph Health institutional review board, with waiver of informed consent. The research reported has adhered to the ethical guidelines set for human subjects research.

## Funding

This research did not receive any specific grant from funding agencies in the public, commercial, or not-for-profit sectors.

## Disclosures Statement

E. C. Korngold: Abbott Vascular: Consulting, honoraria; Boston Scientific: Consulting, honoraria; Edwards Lifesciences: Consulting, honoraria, proctoring; Medtronic: Consulting, honoraria; RJ, none; KJS, none; VK, none; BC, Abbott Vascular: Consulting; SG, Edwards Lifesciences: consultant; DP, none; DS: none; AR: none; LM: none; BJ, none. The other authors had no conflicts to declare.
